# Association of Cold-Heat Patterns with Tongue Features, Body Composition, Anthropometric Indices, and Blood Parameters in Tae-Eum Type

**DOI:** 10.1155/2018/2754195

**Published:** 2018-10-08

**Authors:** Jihye Kim, Soo Jung Park, Jiwon Yoon, Bum Ju Lee, Keun Ho Kim

**Affiliations:** ^1^Future Medicine Division, Korea Institute of Oriental Medicine, 1672 Yuseong-daero, Yuseong-gu, Daejeon, Republic of Korea; ^2^Department of Sasang Constitutional Medicine, College of Korean Medicine, Woosuk University, Republic of Korea

## Abstract

**Introduction:**

The purpose of this study was to elucidate the relationship between cold-heat patterns and body composition, anthropometric indices, blood parameters, and tongue features in Tae-Eum type subjects. We also sought to determine whether significant indicators could be used as risk factors for predicting cold-heat patterns in a clinic.

**Methods:**

This prospective, case-control pilot study was conducted at a single center. The subjects were males and females aged 19 years or older who had been analyzed as the Tae-Eum type. After screening, subject allocation was performed. The body composition, 11 anthropometric indices, blood parameters, and tongue features of the subjects were measured by well-trained practitioners. An independent t-test was conducted to compare the cold- and heat-pattern groups. Binary logistic regression was performed to determine significant differences between the two groups after adjusting for age, sex, and systolic blood pressure, with a focus on identifying significant indicators.

**Results:**

Eighty-nine participants were recruited, 39 of whom were excluded from the analysis. Ultimately, 20 cold-pattern and 30 heat-pattern subjects were included in the final analysis. We found that alanine aminotransferase and all anthropometric indices, except for neck and forehead circumference, were significant predictive factors in both groups according to the binary regression analysis. Additionally, the tongue body color in the cold-pattern group was significantly paler than that in the heat-pattern group.

**Conclusions:**

This study found that cold and heat patterns were significantly associated with body composition, anthropometric indices, blood parameters, and tongue features. We suggest that these factors could thus be used as objective indicators and predictors of cold-heat patterns. Our findings provide fundamental but also applicable data that will be useful for diagnosing and monitoring cold-heat patterns in Tae-Eum type patients.

## 1. Introduction

Sasang constitutional medicine is an exclusive and unique system of traditional Korean medicine (TKM) initiated in the late 19^th^ century by Lee Je-Ma, which adopts a distinctive perspective viewpoint from the traditional Eastern medicine of China or Korea [1, 2]. Sasang constitutional types (SCTs) consist of four classifications: Tae-Yang, Tae-Eum, So-Yang, and So-Eum [3]. These types were diagnosed based on physical, physiological, and psychological characteristics such as susceptibility to particular external conditions or disease and types of weak or strong organ function [3, 4].

In Sasang constitutional medicine, patients diagnosed with one of the four SCTs are treated based on cold or heat patterns induced by conditions of physiological equilibrium among the internal organs, which also induce differences in body circumference [5]. For pattern identification, the most important and unique patterns are the cold-heat patterns, which reflect not only the temperature but also the metabolic activity of the subject [6]. Cold and heat patterns are determined by integrating four diagnostic methods. These patterns play an important role in deciding appropriate treatment regimens with modalities such as herbal prescriptions and acupuncture [7]. The cold pattern is related to low and slow metabolism, whereas the heat pattern is related to increase in the metabolic rate. Therefore, patients with a heat pattern show signs and symptoms such as red complexion, high fever, lots of sweat, thirst, yellow urine, and a rapid pulse. On the other hand, patients with a cold pattern display symptoms including pale complexion, dislike of cold, cold limbs, little sweating and thirst, clear urine, and a slow pulse [8].

Although diagnosing cold-heat patterns is challenging, making an exact diagnosis of cold-heat patterns is very important. Pattern identification results are affected not only by external factors such as temperature, humidity, and luminance but also by subjective factors related to the knowledge, experience, and diagnostic skills of individual practitioners in assessing a patient's pulse, face, tongue, voice, body shape, stool, urine, sweating habits, and skin [9]. Several studies on cold-heat patterns have focused on specific diseases, such as rheumatoid arthritis [10] and the common cold [11], or on the development of cold-heat questionnaires [9, 12, 13]. However, existing studies on SCTs are insufficient, as there are currently no published reports evaluating the differences between cold-heat patterns in specific SCTs in terms of variables such as vital signs, body composition, anthropometric indices, blood parameters, and tongue features. In particular, it is very important to accurately diagnose the cold-heat patterns in Tae-Eum type because not only the cold-heat patterns of Tae-Eum type are very different from the manifestation symptoms but also the prescribed specific medical herbs are strictly different. Tae-Eum type diagnosed as cold pattern uses the following medical herbs: ephedrae herba, coicis semen, castaneae semen, liriopes radix, platycodi radix, and schisandrae fructus. Tae-Eum type diagnosed as heat pattern uses the following medical herbs: puerariae radix, scutellariae radix, cimicifugae rhizoma, angelicae dahuricae radix, rhei rhizoma, and ligustici tenuissimae radix.

Therefore, as a first step, we tried to determine the differences of variables such as vital signs, body composition, anthropometric indices, blood parameters, and tongue features between cold-heat patterns in Tae-Eum type.

The aim of this study was to evaluate the associations of cold-heat patterns in the Tae-Eum type with body composition, anthropometric indices, blood parameters, and tongue features. Additionally, we sought to determine whether significant indicators could be used as risk factors for predicting cold-heat patterns in a clinic.

## 2. Materials and Methods

### 2.1. Study Hypothesis

The hypothesis was that body composition, anthropometric indices, blood parameters, and tongue features would significantly differ between the cold- and heat-pattern groups in Tae-Eum type patients.

### 2.2. Subjects

This study was conducted at a single center as a prospective, exploratory pilot clinical study. The entire study was conducted at the Woosuk University Medical Center (WUMC) in Jeonju, Republic of Korea, between January and March 2017. This study was approved by the Institutional Review Board of WUMC (IRB number WSOH IRB M1709-01-01) and conducted according to the Declaration of Helsinki.

#### 2.2.1. Inclusion and Exclusion Criteria

Subjects were recruited through posters that were displayed around the university and WUMC. The subjects were males and females aged 19 years or older who were diagnosed as the Tae-Eum type. Subjects with one or more of the following criteria were excluded: (1) subjects who had a hypersensitivity reaction after an examination that included a computerized tongue image analysis system; (2) pregnant or breastfeeding women; (3) subjects with a cognitive disorder; (4) subjects who did not sign the informed consent; (5) subjects who were involved in the administration of this research or who were deemed unfit for this research by the administrators; (6) subjects who were diagnosed as non-Tae-Eum types; and (7) subjects who had a fever over 38°C.

#### 2.2.2. Sasang Constitutional Diagnosis

Sasang constitutional integrated diagnostic model developed by Do et al. [14, 15] was used to classify people into four SCTs based on probability values for each type. The model integrates quantitative data from facial images, body shape, voice features, and a questionnaire on personality and physiological symptoms into a single value. The integrated diagnostic model and measurement methods have previously been described in detail. Classification of subjects enrolled in this study was performed by using the final diagnostic model.

#### 2.2.3. Cold-Heat-Pattern Diagnosis

Each subject was diagnosed as having a cold or heat pattern based on the 2015 Sasang constitutional diagnosis guidelines for the Tae-Eum and Tae-Yang types by Lee J et al. In this study, the diagnostic criteria for the cold-heat patterns were ordinary symptoms which means symptoms occurring in association with the individual constitution. The ordinary symptoms of the cold pattern include chilling, no perspiration, headache, backache, myalgia, arthralgia, palpitation, asthma, cough, diarrhea, dyspepsia/postprandial fullness, chest discomfort, and edema. On the other hand, the ordinary symptoms of heat pattern include pyrexia, perspiration, dry eye, dry nose, xerostomia, xeroderma, polydipsia, polyuria, and constipation. A more detailed diagnostic procedure is described in Lee's study [16]. A TKM expert at the hospital with more than 5 years of clinical experience then diagnosed individual cold-heat patterns. Subjects whose expert diagnosis differed from the result produced by the guidelines were excluded from the analysis. For accurate diagnoses, we adhered strictly to the defined qualifications of the expert and subject criteria.

### 2.3. Data Collection

After screening, the enrolled subjects were divided into cold- and heat-pattern groups. After completing the allocation process, the body composition, anthropometric indices, blood parameters, and tongue features of the subjects were obtained for use in a comparative analysis between cold- and heat-pattern groups of Tae-Eum type subjects.

#### 2.3.1. Blood Parameters

A laboratory test was performed to obtain the blood parameters after more than 12 hours of fasting. Total cholesterol (TC), triglycerides (TGs), high-density lipoprotein cholesterol (HDL-C), total protein (TP), albumin, aspartate aminotransferase (AST), alanine aminotransferase (ALT), alkaline phosphatase (ALP), fasting blood glucose (FBG), blood urea nitrogen (BUN), serum creatinine (Crea), calcium (Ca), sodium (Na), potassium (K), chlorides (Cl), white blood cells (WBCs), red blood cells (RBCs), hemoglobin (Hb), platelets, lymphocytes, monocytes, eosinophils, basophils, erythrocyte sedimentation rate (ESR), and C-reactive protein (CRP) were measured at a central laboratory at WUMC.

#### 2.3.2. Body Composition and Anthropometric Indices

Body composition measurements obtained from a body composition analyzer (InBody 720, InBody, Seoul, South Korea) included the following: skeletal muscle mass (SMM), body fat mass (BFM), and body fat percentage (BFP). For the body shape analysis, height, weight, and the following eight circumference indices were collected: forehead circumference, neck circumference, axillary circumference, chest circumference, rib circumference, waist circumference, pelvic circumference, and hip circumference (Table 1). Body mass index (BMI) was simply calculated using a subject's height and weight. Anthropometric parameters were measured by well-trained staff with standard operating procedures (SOPs) developed for the Korea Constitutional Multicenter study based on Sasang constitutional medicine. Body measurement data were collected from each subject while wearing light clothing and maintaining stable breathing [17, 18]. The measurements were performed according to the SOP [17, 18].

#### 2.3.3. Tongue Features

**Figure 1:**
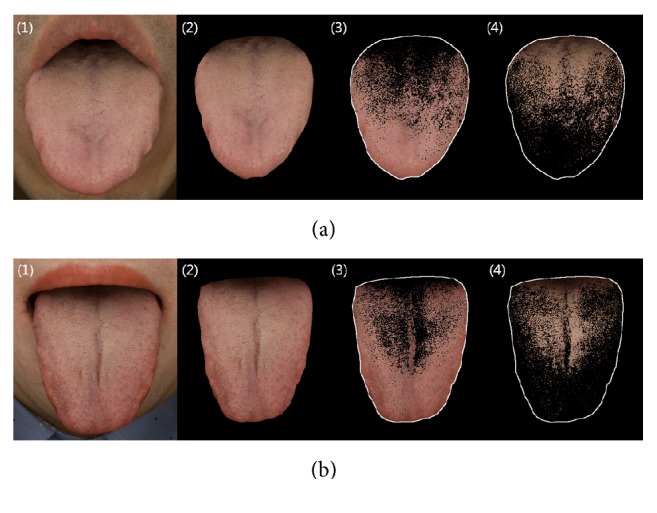
Row (a) presents tongue images from a cold-pattern subject. Row (b) presents tongue images from a heat-pattern subject. Illustrations show (1) the original tongue image, (2) segmented tongue region, (3) tongue substance area, and (4) tongue coating area.

The following tongue features were obtained by using a computerized tongue image analysis system (CTIS; TAS-2000, Korea Institute of Oriental Medicine, Daejeon, South Korea): tongue body color and tongue coating color. The images were taken within 5-10 seconds and the captured images were automatically color-corrected by using a color chart (ColorChecker® Classic, X-Rite Pantone, USA). Afterward, the color-corrected images were semi-automatically segmented into the tongue region without background (Figure 1. (2)), tongue body area (Figure 1 (3)), and tongue coating area (Figure 1. (4)).

Tongue body and coating color values were obtained from the International Commission on Illumination LAB which is usually abbreviated CIE LAB for its French name, Commission Internationale de l'éclairage LAB. The CIE LAB color space, developed in 1976, describes all the colors visible to human eyes and one of the most widely used today. The CTIS components and protocol for image analysis have previously been described in detail [19, 20].

### 2.4. Data Analysis

All analyses were conducted with SPSS version 23.0 (SPSS Inc., Chicago, Illinois, USA). The general characteristics of the participants were analyzed by using analyses of means and standard deviations. An independent *t*-test was conducted to compare the cold and heat groups. In the crude analysis and the analysis adjusted for age, sex, and systolic blood pressure (SBP), binary logistic regression was conducted to identify significant differences between the cold-pattern group (0) and the heat-pattern group (1). Statistical significance was defined as *p* < 0.05.

## 3. Results

### 3.1. General Characteristics

A total of 89 subjects were recruited. Six subjects violated the protocol and were subsequently excluded from the data set. Another 33 subjects who were not diagnosed with cold or heat patterns (e.g., cold-heat complex or non-cold, non-heat) were excluded from the analysis. In total, 50 subjects were assigned to the cold- and heat-pattern groups.

The general characteristics of the participants are presented in Table 2 according to cold-heat-pattern group. The mean age and standard deviation of the cold- and heat-pattern groups were 43.7 ± 16.71 and 47.93 ± 15.56 years, respectively. There were no significant differences in mean age between the two groups. Additionally, there were no significant differences in marital status, occupation, or education. There was a slightly significant difference in sex and a strongly significant difference in SBP between the groups, but there were no significant differences in diastolic blood pressure, pulse, respiration rate or temperature.

### 3.2. Comparison of Body Composition and Anthropometric Indices between Cold- and Heat-Pattern Groups

The body composition data and anthropometric indices of the cold- and heat-pattern groups are presented in Table 3. All indices were significantly higher in the heat group than in the cold group, except for BFP and height. BFP and height did not differ between the two groups (*p* = 0.342 and *p* = 0.105, respectively).

### 3.3. Comparison of Blood Parameters between Cold- and Heat-Pattern Groups

Table 4 shows the comparison of blood parameters between the cold- and heat-pattern groups. There were no significant differences in the levels of TC, TP, albumin, AST, ALP, FBG, BUN, Crea, Ca, Na, K, Cl, WBCs, platelets, segmented neutrophils, lymphocytes, monocytes, eosinophils, basophils, ESR, or CRP between the two groups. However, there was a strongly significant difference in mean ALT and Hb between the groups (*p* ≤ 0.001 and *p* = 0.009, respectively). There were also weak significant differences in the levels of TGs, HDL-C, and RBCs between the groups (*p* = 0.026, *p* = 0.027, and *p* = 0.011, respectively).

### 3.4. Comparison of Tongue Features between Cold- and Heat-Pattern Groups

The tongue features of the cold-pattern and heat-pattern groups are presented in Table 5. Based on the CIEL^*∗*^a^*∗*^b^*∗*^ model, the CIE L^*∗*^ value for tongue body area was higher in the cold-pattern group than in the heat-pattern group (48.23 ± 3.70 versus 45.76 ± 2.56, *p* = 0.008). No differences in the CIE a^*∗*^ and b^*∗*^ values for tongue body area were found between the two groups.

Additionally, there was no difference in the CIE L^*∗*^ value for tongue coating area between the groups. However, the CIE a^*∗*^ and b^*∗*^ values for tongue coating area were significantly higher in the cold-pattern group than in the heat-pattern group (*p* = 0.012 and *p* = 0.047, respectively).

### 3.5. Associations between Cold-Heat Pattern and Body Composition, Anthropometric Indices, Blood Parameters, and Tongue Features

The associations between the significant indicators and the cold-heat pattern are shown in Table 6. SMM was significantly associated with cold-heat pattern in the crude analysis (*p* = 0.004, OR = 1.196 [1.059-1.351]) and after adjusting for age, sex, and SBP (adjusted *p* = 0.021, adjusted OR = 1.338 [1.045-1.713]). BFM was not associated with cold-heat pattern (*p* = 0.337, OR = 1.044 [0.956-1.140]) in the crude analysis, but there was a significant association after adjusting for confounders (adjusted *p* = 0.020, adjusted OR = 1.248 [1.036-1.504]).

Of the anthropometric indices, weight and BMI were significantly associated with cold-heat pattern in both the crude (*p* = 0.001, OR = 1.171 [1.065-1.288] and 1.840 [1.290-2.626]), respectively) and adjusted analyses (adjusted *p* = 0.006, adjusted OR = 1.184 [1.050-1.336]; adjusted *p* = 0.003, adjusted OR = 2.100 [1.290-3.419], respectively). Cold-heat pattern was not related to forehead circumference in either the crude or adjusted analyses (*p* = 0.054, OR = 1.458 [0.994-2.138]; adjusted *p* = 0.399, adjusted OR = 1.265 [0.733-2.182]). Neck circumference was significantly associated with cold-heat pattern in the crude analysis (*p* = 0.020, OR = 1.295 [1.042-1.610]), but this association did not remain after adjusting for confounders. Axillary, chest, rib, waist, pelvic, and hip circumferences were associated with cold-heat pattern in both the crude and adjusted analyses.

Among the blood parameters, TG levels, RBCs, and Hb were associated with cold-heat pattern only in the crude analysis (*p* = 0.043, OR = 1.007 [1.000-1.015]); *p* = 0.019, OR = 6.937 [1.382-34.81]; *p* = 0.018, OR = 1.721 [1.098-2.697], respectively). HDL-C was significantly associated with cold-heat pattern in the crude analysis (*p* = 0.035, OR = 0.937 [0.882-0.995]), but this association did not remain significant after adjusting for confounders (*p* = 0.057, OR = 0.927 [0.856-1.002]). ALT was associated with cold-heat pattern in both the crude (*p* = 0.004, OR = 1.086 [1.027-1.148]) and adjusted analyses (adjusted *p* = 0.035, adjusted OR = 1.066 [1.005-1.131]).

Finally, for the tongue features, cold-heat pattern was associated with the CIE L^*∗*^ value for tongue body (*p* = 0.016, OR = 0.763 [0.611-0.951]) and the CIE a^*∗*^ value for tongue coating (*p* = 0.020, OR = 0.274 [0.092-0.814]) in the crude analysis, but the association between cold-heat pattern and CIE L^*∗*^ was no longer significant after adjusting for confounders (adjusted *p* = 0.088, adjusted OR = 0.808 [0.633-1.032]). Additionally, the CIE b^*∗*^ value for tongue coating was not related to cold-heat pattern in either the crude or adjusted analyses (*p* = 0.054, OR = 0.746 [0.554-1.005]; adjusted *p* = 0.155, adjusted OR = 0.767 [0.531-1.106]).

## 4. Discussion

In the present study, a comparative analysis was performed to examine differences in body composition, anthropometric indices, blood parameters, and tongue features between cold- and heat-pattern groups in Tae-Eum type patients.

The results showed that heat-pattern subjects presented a more reddish tongue body and were more obese than cold-pattern ones.

From the *t*-test and binary logistic regression analyses, we found that the body composition factors SMM and BFM as well as the anthropomorphic indices like weight, BMI, axillary, chest, rib, waist, pelvic, and hip circumferences were highly associated with the heat pattern in both the crude and adjusted analyses, although BFM was not significant in the adjusted analysis. There was a positive correlation between the heat pattern and the above anthropometric indices. Yoon J et al. observed that the indicators of body water balance (extracellular water/intracellular water ratio and extracellular water/total body water ratio) and Sasang personality questionnaire scores were significantly different between the So-Yang and non-So-Yang types [21]. Additionally, Jang E et al. evaluated whether SCTs could be a risk factor for abdominal obesity in Korean populations. The results of that study showed that, after adjustment, the Tae-Eum type was associated with increased abdominal obesity prevalence compared to the So-Eum and So-Yang types among both males and females [22]. Kim BS et al. compared differences in gut microbiota among three constitutions (So-Yang, So-Eum, and Tae-Eum) where Tae-Yang is excluded in the analysis due to the rare population. They also measured anthropometric and biochemical parameters. The results showed that height, weight, BMI, waist circumference, lean body mass, fat mass, and fat percentage differed significantly among the three types [23]. Park YJ et al. reported that there were no significant differences in age, height, weight, or BMI between cold- and heat-prescription groups in male and female Tae-Eum types [24]. According to Sasang constitutional medicine theory, lung hypofunction and liver hyperfunction are related to a large WC, and the Tae-Eum type is associated with hyperactive liver function and a trim waist area [25]. As mentioned above, various studies have investigated associations between Sasang constitutional medicine and BMI or the indices related to abdominal obesity. According to many previous studies, the prevalence and relative risk of obesity and metabolic syndrome are higher in the Tae-Eum type than in other types [26]. However, as there are currently no studies comparing body composition and anthropometric indices between cold- and heat-pattern patients of specific SCT types, the results of the present study are important.

In the present analysis of blood parameters, only ALT was significant in both the crude and adjusted analyses. Kim KY et al. found significant correlations between the Tae-Eum type and blood parameters such as hematocrit, TP, TGs, phospholipids, TC, low-density lipoprotein cholesterol, BUN, and cortisol (*p* = 0.05). Furthermore, Jang E et al. found that SCTs were significantly correlated with FBG, TG, and HDL-C (*p* < 0.001) [22]. Kim BS et al. compared blood parameters among the So-Yang, So-Eum, and Tae-Eum types and found that there were no significant differences in FBG, TGs, HDL-C, or TC among the three groups [23]. Park et al. compared 7 blood parameters, including ALT, TGs, WBCs, Hb, AST, and TC, between cold- and heat-pattern in Tae-Eum type and found that there were significant differences in ALT, TGs, WBCs, and Hb, but not AST and TC, between the two groups [27].

In the present study, we also found that the tongue body color of the cold-pattern subjects was significantly paler than that of the heat-pattern subjects. According to traditional medicine theory, changes in the tongue features indicate both the state of organ function and imbalances in essential components, allowing the doctor to determine whether the patient has a heat or cold pattern [28–30]. Patients with a typical cold pattern normally exhibit a pale tongue and thin tongue coating, whereas heat-pattern patients normally exhibit a reddish tongue and thick fur. Interestingly, the results of this study are consistent with TKM theory [30]. Although there are no previous studies examining tongue color, a comparative study was conducted on the facial color of patients with cold and heat patterns in the Tae-Eum type by Park YJ et al. [31]. They observed that facial colors based on actual clinical data were similar among three types (Tae-Eum, So-Yang, and So-Eum). The results showed significant differences in complexion between the cold- and heat-prescription groups, which demonstrated that patterns differed according to Sasang constitution [29]. Since it is easier to observe the health condition in the tongue than in the face, it is possible to develop an objective diagnostic index that can distinguish the cold and heat patterns in Tae-Eum type patients with a tongue if sufficient in-depth research is conducted. Therefore, we suggest that the tongue body and coating colors should be used as a diagnostic and monitoring indicator for identifying cold and heat patterns in Tae-Eum type patients.

This study had two limitations. This study was designed as a single-center, prospective, cross-sectional pilot study. Therefore, it may be difficult to confirm the final results of the analysis. We aim to conduct a larger, multicenter trial after further considering the results of this study. Second, this study lacked observations regarding the relationships between other SCT types and body composition, anthropometric indices, blood parameters, and tongue features. This study was a preliminary study focused only on the Tae-Eum type. The results of this study show that it is possible to estimate cold and heat patterns in Tae-Eum type patients. Having confirmed this possibility, we plan to conduct a research on other SCT types in the future. Third, the difference between cold-heat patterns in the Tae-Eum type includes both the exterior and interior symptomatology of Tae-Eum type, as well as the nature and mind. This study lacks observations regarding the association of nature and mind between cold-heat patterns in Tae-Eum type. Further research should be conducted to overcome these limitations. Despite its limitations, this study was the first attempt to explore differences in body composition, anthropometric indices, blood parameters, and tongue features between cold- and heat-pattern patients of a specific SCT. Although this was a pilot study, the results represent valuable and important basic information. This analysis may provide fundamental but also applicable data that will be useful for diagnosing and monitoring cold-heat patterns in Tae-Eum type patients.

## 5. Conclusion

This study aimed to elucidate the relationship between cold-heat pattern and body composition, anthropometric indices, blood parameters, and tongue features among Tae-Eum type patients. It is very important to correctly diagnose cold and heat patterns after a patient's SCT type has been identified. We found significant differences in body composition, anthropometric indices, blood parameters, and tongue features between the cold- and heat-pattern groups in Tae-Eum type patients. These findings are consistent with previous studies and SCM theory. Thus it is believed that these indices may serve as elementary and supplementary means for the differentiation of syndromes in Tae-Eum type. We need to continue this research in a larger, multicenter trial after further consideration of the results of this study.

## Figures and Tables

**Table 1:** Definitions of anthropometric indices.

Anthropometric index	Definition
Height (cm)	Distance from the top of the subject to the bottom
Weight (kg)	Mass of the subject
Body mass index (kg/cm^2^)	Body mass divided by body height squared
Forehead circumference (cm)	Levels of the glabella and opisthion
Neck circumference (cm)	Thyroid cartilage and cricoid cartilage
Axillary circumference (cm)	Left and right axilla
Chest circumference (cm)	Left and right nipple points
Rib circumference (cm)	Left and right 7th and 8th prominence of costochondral junction
Waist circumference (cm)	Umbilical cord
Pelvic circumference (cm)	Left and right anterior superior iliac spines
Hip circumference (cm)	Upper edge of the pubis

**Table 2:** General characteristics of cold- and heat-pattern groups (n = 50).

Variables	Cold pattern (n = 20)	Heat pattern (n = 30)	*p*-value
Age (mean, SD)	43.7 (16.71)	47.93 (15.56)	0.365
Sex (no. of subjects, %)^†^			0.028
Male	7 (35.0)	20 (66.7)	
Female	13 (65.0)	10 (33.3)	
Vital signs			
Systolic blood pressure (mmHg)^‡^	121.4 (12.63)	131.7 (12.64)	0.007
Diastolic blood pressure (mmHg)	81.9 (9.94)	86.2 (10.47)	0.153
Pulse (beats/min)	77.55 (9.1)	78.47 (8.7)	0.722
Respiration rate (/min)	17.5 (2.24)	17.2 (2.76)	0.687
Temperature (°C)	36.66 (0.16)	36.64 (0.21)	0.676
Marital status (no. of subjects, %)			0.644
Single	9 (45.0)	10 (33.3)	
Married	10 (50.0)	19 (63.3)	
Other	1 (5.0)	1 (3.3)	
Occupation (no. of subjects, %)			0.183
White-collar worker	1 (5.0)	6 (20.0)	
Office worker	1 (5.0)	2 (6.7)	
Service	3 (15.0)	6 (20.0)	
Sales	3 (15.0)	0 (0.0)	
Blue-collar worker	0 (0.0)	1 (3.3)	
Other	12 (60.0)	15 (50.0)	
Education (no. of subjects, %)			0.599
Elementary school	0 (0.0)	3 (10.0)	
Middle school	2 (10.0)	2 (6.7)	
High school	9 (45.0)	12 (40.0)	
University	8 (40.0)	10 (33.3)	
Graduate school	1 (5.0)	3 (10.0)	

^†^ *p* < 0.05; ^‡^*p* < 0.01.

**Table 3:** Comparison of body composition and anthropometric indices between cold- and heat-pattern groups (n = 50).

Variables	Cold pattern (n = 20)	Heat pattern (n = 30)	*p*-value
Body composition			
Skeletal muscle mass^‡^	25.48 (4.89)	31.26 (6.31)	0.001
Body fat mass^‡^	18.78 (4.71)	24.97 (6.93)	0.001
Body fat percentage (%)	29.01 (6.89)	30.86 (6.55)	0.342
Anthropometric indices			
Height (cm)	163.34 (8.29)	167.25 (8.14)	0.105
Weight (kg)^‡^	65.16 (7.75)	80.77 (13.43)	0.001
Body mass index (kg/cm^2^)^‡^	24.42 (2.32)	28.74 (3.16)	0.001
Forehead circumference (cm)^†^	56.22 (1.69)	57.18 (1.59)	0.046
Neck circumference (cm)^†^	35.20 (3.23)	37.39 (2.70)	0.013
Axillary circumference (cm)^‡^	91.85 (4.57)	100.70 (6.74)	0.001
Chest circumference (cm)^‡^	93.60 (5.04)	101.60 (7.33)	0.001
Rib circumference (cm)^‡^	81.49 (6.61)	93.02 (7.44)	0.001
Waist circumference (cm)^‡^	85.06 (6.59)	94.41 (8.55)	0.001
Pelvic circumference (cm)^‡^	89.94 (8.21)	97.57 (7.52)	0.001
Hip circumference (cm)^‡^	97.01 (4.00)	101.97 (6.37)	0.001

^†^ *p* < 0.05; ^‡^*p* < 0.01.

**Table 4:** Comparison of blood parameters between cold- and heat-pattern groups (n = 50).

Variables	Cold pattern (n = 20)	Heat pattern (n = 30)	*p*-value
Total cholesterol (mg/dL)	189.05 (47.89)	205.53 (40.13)	0.194
Triglycerides (mg/dL)^†^	131.3 (84.36)	209.97 (136.71)	0.026
High-density lipoprotein (mg/dL)^†^	55.7 (12.19)	48.97 (8.64)	0.027
Total protein (g/dL)	7.46 (0.37)	7.46 (0.36)	0.975
Albumin (g/dL)	4.73 (0.26)	4.73 (0.3)	0.968
Aspartate aminotransferase (U/L)	24.75 (10.05)	27.4 (9.39)	0.346
Alanine aminotransferase (U/L)^‡^	20.45 (11.93)	37.2 (17.61)	0.001
Alkaline phosphatase (U/L)	27.9 (24.33)	38.17 (25.57)	0.163
Fasting blood glucose (mg/dL)	100.1 (26.54)	110.4 (43.99)	0.353
Blood urea nitrogen (mg/dL)	14.19 (3.89)	14.89 (3.67)	0.521
Creatinine (mg/dL)	0.87 (0.19)	0.93 (0.16)	0.198
Calcium (mg/dL)	8.97 (0.34)	8.98 (0.26)	0.829
Sodium (mmol/L)	138.65 (1.66)	138.23 (1.41)	0.345
Potassium (mmol/L)	4.21 (0.28)	4.35 (0.32)	0.116
Chloride (mmol/L)	102.65 (1.57)	102.57 (2.1)	0.880
White blood cells (K/uL)	6.36 (1.58)	7.17 (1.69)	0.096
Red blood cells (M/uL)^†^	4.6 (0.41)	4.92 (0.44)	0.011
Hemoglobin (g/dL)^†^	13.65 (1.88)	14.86 (1.25)	0.009
Platelets (K/dL)	249.8 (51.61)	250.23 (69.74)	0.981
Segmented neutrophils (%)	52.65 (6.81)	52.97 (8.24)	0.887
Lymphocytes (%)	35.95 (4.86)	36.13 (6.83)	0.918
Monocytes (%)	7.7 (2.18)	7.67 (1.97)	0.955
Eosinophils (%)	3.25 (3.37)	2.83 (2.07)	0.590
Basophils (%)	0.45 (0.51)	0.4 (0.5)	0.732
Erythrocyte sedimentation rate (mm/h)	6.45 (6.05)	5.17 (6.06)	0.466
C-reactive protein (mg/L)	1.44 (1.35)	1.73 (1.62)	0.510

^†^ *p* < 0.05; ^‡^*p* < 0.01.

**Table 5:** Comparison of tongue features between cold- and heat-pattern groups (n = 50).

Variables	Cold pattern (n = 20)	Heat pattern (n = 30)	*p*-value
Tongue body area			
CIE L^‡^	48.23 (3.7)	45.76 (2.65)	0.008
CIE a	21.49 (1.56)	22.27 (1.03)	0.059
CIE b	15.81 (2.47)	14.78 (1.68)	0.114
Tongue coating area			
CIE L	39.9 (7.25)	36.94 (6.51)	0.139
CIE a^†^	13.48 (0.55)	13.02 (0.66)	0.012
CIE b^†^	14.66 (2.18)	13.45 (1.98)	0.047

^†^ *p* < 0.05; ^‡^*p* < 0.01.

**Table 6:** Associations between cold-heat pattern and body composition, anthropometric indices, blood parameters, and tongue features.

Cold pattern (reference)	Heat pattern			
	Crude		Adjusted	
	OR (95% CI)	*p*-value	OR (95% CI)	*p*-value
Skeletal muscle mass	1.196 (1.059-1.351)	0.004	1.338 (1.045-1.713)	0.021
Body fat mass	1.044 (0.956-1.140)	0.337	1.248 (1.036-1.504)	0.020
Weight	1.171 (1.065-1.288)	0.001	1.184 (1.050-1.336)	0.006
Body mass index	1.840 (1.290-2.626)	0.001	2.100 (1.290-3.419)	0.003
Forehead circumference	1.458 (0.994-2.138)	0.054	1.265 (0.733-2.182)	0.399
Neck circumference	1.295 (1.042-1.610)	0.020	1.159 (0.860-1.562)	0.332
Axillary circumference	1.394 (1.147-1.693)	0.001	1.410 (1.113-1.785)	0.004
Chest circumference	1.266 (1.094-1.465)	0.002	1.316 (1.087-1.594)	0.005
Rib circumference	1.300 (1.116-1.514)	0.001	1.461 (1.143-1.868)	0.002
Waist circumference	1.194 (1.065-1.339)	0.002	1.180 (1.034-1.346)	0.014
Pelvic circumference	1.144 (1.041-1.257)	0.005	1.197 (1.060-1.353)	0.004
Hip circumference	1.210 (1.047-1.398)	0.010	1.197 (1.023-1.401)	0.025
Triglycerides	1.007 (1.000-1.015)	0.043	1.005 (0.998-1.012)	0.140
High-density lipoprotein	0.937 (0.882-0.995)	0.035	0.927 (0.856-1.002)	0.057
Alanine aminotransferase	1.086 (1.027-1.148)	0.004	1.066 (1.005-1.131)	0.035
Red blood cells	6.937 (1.382-34.81)	0.019	4.649 (0.523-41.29)	0.168
Hemoglobin	1.721 (1.098-2.697)	0.018	1.601 (0.792-3.233)	0.190
CIE L^*∗*^ value of tongue body	0.763 (0.611-0.951)	0.016	0.808 (0.633-1.032)	0.088
CIE a^*∗*^ value of tongue coating	0.274 (0.092-0.814)	0.020	0.232 (0.059-0.916)	0.037
CIE b^*∗*^ value of tongue coating	0.746 (0.554-1.005)	0.054	0.767 (0.531-1.106)	0.155

Results of binary logistic regression. OR: odds ratio, adjusted for age, sex, and systolic blood pressure.

## Data Availability

The clinical data collected at the Woosuk University Medical Center used to support the findings of this study are available from the corresponding author upon request.
